# Risk factors for respiratory illness in a community of wild chimpanzees (*Pan troglodytes schweinfurthii*)

**DOI:** 10.1098/rsos.180840

**Published:** 2018-09-19

**Authors:** Melissa Emery Thompson, Zarin P. Machanda, Erik J. Scully, Drew K. Enigk, Emily Otali, Martin N. Muller, Tony L. Goldberg, Colin A. Chapman, Richard W. Wrangham

**Affiliations:** 1 Department of Anthropology, University of New Mexico, Albuquerque, NM, USA; 2 Kibale Chimpanzee Project, Fort Portal, Uganda; 3 Department of Anthropology, Tufts University, Medford, MA, USA; 4 Department of Human Evolutionary Biology, Harvard University, Cambridge, MA, USA; 5 Department of Pathobiological Sciences and Global Health Institute, University of Wisconsin-Madison, Madison, WI, USA; 6 Department of Anthropology, McGill University, Montreal, Quebec, Canada

**Keywords:** disease, immunocompetence, apes, human–wildlife interaction, epidemiology, ageing

## Abstract

Respiratory illnesses have caused significant mortality in African great ape populations. While much effort has been given to identifying the responsible pathogens, little is known about the factors that influence disease transmission or individual susceptibility. In the Kanyawara community of wild chimpanzees, respiratory illness has been the leading cause of mortality over 31 years, contributing to 27% of deaths. Deaths were common in all age groups except juveniles. Over 22 years of health observations, respiratory signs were rare among infants and most common among older adults of both sexes. Respiratory signs were also common among males during the transition to adulthood (ages 10–20 years), particularly among those of low rank. Respiratory signs peaked conspicuously in March, a pattern that we could not explain after modelling climatic factors, group sizes, diet or exposure to humans. Furthermore, rates of respiratory illness in the chimpanzees did not track seasonal rates of illness in the nearby village. Our data indicate that the epidemiology of chimpanzee respiratory illness warrants more investigation but clearly differs in important ways from humans. Findings on individual susceptibility patterns suggest that respiratory signs are a robust indicator for investigating immunocompetence in wild chimpanzees.

## 1. Introduction

Recent reports from across Africa (table 1) suggest that epidemics of infectious disease can have profound impacts on chimpanzee populations [1], with respiratory illness implicated in approximately 18 deaths during 3 outbreaks at Mahale, Tanzania [2,3], 17 deaths during 3 outbreaks at Taï, Côte d'Ivoire [1], 5–6 individuals during 2 outbreaks at Bossou, Guinea [4], at least 28 deaths during 7 outbreaks at Gombe, Tanzania [5,6], 5 deaths during one recent outbreak at Kanyawara, Uganda [7] and several during an outbreak at Ngogo, Uganda [8]. Although the aetiologies of these outbreaks often remain unknown, metapneumovirus (MPV), respiratory syncytial virus (RSV) and rhinovirus-C (RV-C) have been detected in affected individuals [2,7,9], with co-infection by *Streptococcus* or *Pasteurella* bacteria in several lethal cases [1,9–12]. Respiratory illness is a common cause of morbidity and mortality in captive apes, as well, with all species exhibiting high prevalence (greater than 70%) of antibodies to RSV [13].

**Table 1.** Documented respiratory epidemics with more than one death in wild chimpanzee communities.

				mortality (*N*)^b^		
site	year	pathogen^a^	morbidity (%)	adults/adolescents	juveniles	infants
Gombe [5]	1968		63	4	0	0
Gombe [5]	1987		25	9	0	0
Gombe [6]^c^	1996		∼100	5	1	2
Gombe [5]^c^	2000	*SPn*/*Spy*	73	2	0	0
Taï [1]	1999	hRSV/*SPn*	100	5	1	0
Taï [1]	2004	hMPV/*PMc*	100	0	3	5
Taï [1]	2006	hRSV/*SPn*	92	2	0	0
Mahale [2]	2003		98	0	0	4
Mahale [2]	2005		52	0	0	2
Mahale [2,3]	2006	hMPV	48	4	1	7
Bossou [4]	2011		100	3	0	2
Kanyawara [7]^d^	1998		45	3	0	0
Kanyawara [7]^d^	2006		82	1	0	1
Kanyawara [7]^d^	2013	RV-C	87	4	0	1
*total*				*42*	*6*	*24*

^a^*SPn*: *Streptococcus pneumoniae*; *SPy*: *Streptococcus pyogenes*; hRSV: human respiratory syncytial virus; hMPV: human metapneumovirus; *PMc*: *Pasteurella multocida*; RV-C: rhinovirus-C.

^b^Age categories were not always defined in previous studies so are only approximately comparable; totals do not include infants orphaned by mothers infected with the respiratory illness.

^c^Additional details from E. V. Lonsdorf 2017, personal communication.

^d^This study.

Molecular analyses of RSV, MPV and RV-C indicate that the viruses found in chimpanzees are reverse zoonoses (i.e. originate from humans), although it is unclear whether successive outbreaks arise from reinfections by local human populations or single introduction events followed by chimpanzee-to-chimpanzee transmission [2,7,9,14]. While considerable research effort has been put into describing the pathogens themselves, the factors affecting susceptibility to infection and mortality have barely been examined. The only studies to do so examined the incidence of respiratory signs at Gombe National Park over two periods in which no major outbreaks occurred [15,16]. Respiratory signs in Gombe were more frequent in the dry season compared to the wet season [15]. While feeding on provisioned bananas predicted respiratory signs, other exposures to humans (follow time, proximity to research camp) did not, nor did group size or exposure to baboons [15]. Individualistic factors, such as age, sex, dominance rank and SIVcpz infection status did not predict rates of respiratory signs [16]. Across all reports of chimpanzee respiratory outbreaks, several outbreaks have caused mortality only in infants, whereas others have disproportionately killed older individuals (table 1).

In this paper, we report mortality from respiratory illness over 31 years in the Kanyawara community of wild East African chimpanzees living in the Kibale National Park, Uganda. We additionally investigate demographic and temporal predictors of respiratory signs over 22 years of health monitoring. We predicted that rates of respiratory morbidity and mortality would be relatively high among individuals with naive or senescent immune systems—the very young and old—and those with potential energetic disadvantages (low-ranking individuals, pregnant females and those nursing young infants). We predicted that climatic factors (rainfall, temperature) would affect respiratory signs, as they do in human populations [17]. Given the human origin of chimpanzee respiratory infections identified to date, we predicted that exposure to humans and human settlements would lead to temporal increases in respiratory signs, as would the size of chimpanzee aggregations, increasing transmission between individuals. Finally, we predicted that temporal variation in respiratory signs among the chimpanzees would correspond to the rates of human respiratory symptoms reported in the nearby villages, alone or in interaction with the number of human observers to which the chimpanzees were exposed.

## 2. Material and methods

Data were recorded on the Kanyawara community of chimpanzees in Kibale National Park, Uganda. This long-term study was established by Wrangham in 1987 [18], and thereafter chimpanzees were followed on a near-daily basis. Chimpanzee mortalities reported here are from the full study period of 1987–2017, during which time the community size varied from 45 to 55 individuals. Deaths were attributed following positive identification of carcasses or the disappearance of regularly observed individuals. Females typically transfer between communities at adolescence, thus disappearance of apparently healthy females between the ages of 9 and 15 was not counted in mortality records. Additionally, three mothers and their offsprings were observed too rarely to know whether they were permanent members of the Kanyawara community, thus their disappearances were not attributed to mortality events.

We analysed systematic health records collected between January 1995 and December 2016. On a daily basis, a team of observers (two to six people) collaborated to record the occurrence of clinical signs for all individuals observed that day. These included coughing, sneezing, diarrhoea, unhealed wounds, limping and other less common signs, such as skin abnormalities. During meetings with field directors (R.W.W., M.N.M., M.E.T., Z.P.M. and E.O.) approximately two to three times per year, health definitions were reviewed, and new observers underwent a six-month probationary training and evaluation period by a senior field assistant. In addition to a basic code (presence/absence) for these categories, observers typically recorded a description (e.g. ‘dry cough, only once’ or ‘wet cough, coughing very seriously’). Sneezing and dry coughs were regular occurrences, but were usually isolated (i.e. an individual coughed or sneezed only once) and unlikely to be consequential. When coughing was widespread or frequent, it was almost always described as ‘wet’ or ‘productive’, meaning that it sounded as mucus was present in the respiratory tract, as if the individual were trying to cough something up. Thus, we considered individuals to have *respiratory signs* if they were noted to have (i) a ‘wet’ or ‘productive’ cough; (ii) coughing and sneezing on the same day; (iii) a runny nose; or (iv) frequent or severe coughing or sneezing (e.g. ‘coughing very badly’ or ‘coughing all day’). Chimpanzees exhibit flexible grouping behaviour and range over large areas [19,20], meaning that not every individual can be observed each day. To avoid problems of non-independence, we recorded for each individual and each month of the study (i.e. chimpanzee-month) a dichotomous variable indicating whether the individual was observed on at least 1 day with respiratory signs. If individuals were asymptomatic but not observed for at least 25 h in the month (during observations at the party level), they were excluded from the analysis. The final sample included 120 individuals and 8046 chimpanzee-months (mean = 67, range 1–246 months per individual). For the purposes of calculating incidence rates, we defined a unique case as a month in which respiratory signs were observed but signs had not been observed in the previous month. This may slightly underestimate true prevalence rates if respiratory infections were to occur in rapid succession.

Adult females were classified into four categories based on reproductive profiles: cycling, pregnant, early lactation (first 2 years postpartum), late lactation (from 2 years postpartum until the resumption of cycling). For most females, pregnancy was inferred by back-calculating 225 days from the date of birth and assigning conception to the end of the nearest sexual swelling period [21,22]. In some cases, additional information was available to modify these dates based on pregnancy testing or hormonal analysis [23].

### 2.1. Climatic data

Data on rainfall and temperature were obtained from a weather station located at the Makerere University Biological Field Station within the home range of the Kanyawara chimpanzees. Data were synthesized as monthly values for total rainfall and mean daily minimum and maximum temperature. In three cases, monthly values were unavailable and were thus substituted with the average for that calendar month across the other years.

### 2.2. Behavioural data

We examined four behavioural measures to evaluate their effects on respiratory signs: dietary quality, crop feeding, party size and dominance rank. Chimpanzees eat a variety of foods but prefer ripe fruits, and ripe fruit consumption is a good proxy for nutritional quality of the diet across seasons and the energy balance of chimpanzees [24–26]. Chimpanzee diets were recorded during 15-min scan samples to indicate whether chimpanzees were feeding, and if so, on what food species and part. Dietary quality was quantified as the percentage of feeding observations in which ripe fruit was being consumed during each month (mean = 64%, range 8–91%). This is a global measure of ripe fruit consumption for all observations of chimpanzees that corresponds well with measures derived from individuals [27,28].

Crop feeding was used as an approximation for exposure to humans. Chimpanzees are observed to consume a variety of domesticated foods during excursions to nearby farms: bananas, papaya, maize, sugarcane, guava and sorghum. We defined crop feeding in a similar fashion to ripe fruit feeding, as a percentage of all monthly feeding observations comprising domesticated foods (mean = 2%, range 0–30%). Although not all chimpanzees engage in these excursions, the fact that some individuals do so and then associate with others suggests an epidemiologically plausible mode by which a human-origin respiratory pathogen might be introduced to the chimpanzees.

Chimpanzees are usually found in small subgroupings (parties) of the total community that shift in size and composition over time, and individuals differ in their social preferences and general gregariousness [19,20], suggesting that temporal and individual differences in sociality might impact disease transmission. Records of party size and composition were collected during 15-min scan samples. For each chimpanzee and month, we calculated an individualized measure of mean party size based on the average number of associates (all ages) across all observations of that individual.

Dominance ranks were calculated based on the direction of formal dominance signals (pant-grunt vocalizations) and decided agonistic interactions, including contact aggression, chases and targeted displays. Dominance ranks were generated by using the Elo method [29,30], which provides a cumulative scoring of rank based on each successive interaction and the ratings of competitors. We defined independent events as those occurring at least 10 min apart and set the constant *k*, used to weight outcomes, at 20. We assigned an initial score of 0 to all individuals that were adult at the start of the dataset. Females were subsequently added on the date that they reached sexual maturity (first fully tumescent swelling), and males were added on the date of their first decided win against an adult male, or age 16.5 years, whichever came first. Elo scores for all adults were generated for each day, updating for new interactions. We converted these to ordinal ranks and calculated monthly average rank for each individual. Ranks were then scaled to the number of individuals in the hierarchy (such that 1 = alpha, 0 = omega). Male interactions were frequent enough that we were able to use the Elo method to generate ranks across the entire period 1995–2016. Dominance interactions among female chimpanzees are far less frequent, but relative rankings are highly stable over time [31,32]. Given the rarity of female interactions, it was only possible to assign Elo ratings to all females beginning in 2005. For the years 1997–2004, we calculated female rank scores using the probabilistic scaling method of Jameson *et al*. [33], which also weights interactions based on the strength of opponents but does so using an aggregate dominance matrix. We calculated matrices separately for 1997–2000 and 2001–2004, time periods that were long enough to allow for a sufficient density of interactions but not so long that they included individuals who could not have interacted. These ordinal dominance rankings for 1997–2004 were subsequently standardized and used in the analysis in the same way as ordinal ratings obtained using the Elo method for 2005–2016. Three adult females could not be ranked in one or the other time period because they had no interactions. No female dominance data were available for 1995–1996.

### 2.3. Human data

For rates of human respiratory disease, we obtained data from a clinic in the village of Kanyawara. Established in 2007 as part of a conservation effort for the park, the clinic provides direct health treatment at a greatly subsidized cost to the local community, as well as organizes community-wide vaccinations, de-worming and family planning events [34]. This community directly borders the home range of the chimpanzees, and most legal users of the park either live there or pass through the community to enter the park. The clinic saw an average of 97 visitors per month (range 11–197), and we calculated the percentage that presented with respiratory symptoms, even if that was not their primary complaint. As patients sought clinic services voluntarily, this was not an unbiased estimate but should capture the major seasonal trends. In the same model, we considered the number of unique visitors to the chimpanzees per month, including all field assistants, researchers, students, veterinarians and others, which were recorded on daily field records. Some aggregate values occurred in the records (e.g. ‘film crew’) and were considered to comprise four individuals.

### 2.4. Statistical analysis

Our statistical analysis of respiratory signs comprised two stages. First, we investigated individualistic predictors of respiratory signs (three models: all individuals, adult males, adult females), controlling for variation over time with a fixed categorical effect for a calendar month and a random effect for study year. Second, we attempted to describe the temporal variation by removing the categorical predictors for month and year and replacing them with hypothesized continuous predictors.

All models were generalized linear mixed models (GLMMs) with a binomial distribution and logit link function where the outcome variable was presence/absence of respiratory signs. Each chimpanzee-month comprised a data record. The initial model included all chimpanzee-months (*N* = 8046) and specified fixed effects for age, sex, calendar month and age by sex interaction. To allow for the possibility of nonlinear effects, we explored using quadratic effects of age or age class as a categorical predictor, but model fits were stronger when age was characterized as a simple linear predictor. Two additional terms in the model were included to address potential comorbidities: whether the individual was seen in the same month with diarrhoea or with a fresh wound. We also included a term for observation hours of the target individual during the month, and because a single respiratory illness might extend across months, whether they were seen with signs during the previous month. If a chimpanzee was not observed in the previous month (15.7% of records), we assumed it had not been affected. We also included a random intercept for individual and for a year, entered as a nominal variable to avoid covariation with age. A random slope on the age term was considered but omitted because it explained almost zero variance (0.0000002), leading to convergence problems.

In the second pair of models, we examined the effects of dominance rank on the probability of exhibiting respiratory signs for adult males and the effects of both dominance rank and reproductive state for females, retaining the significant predictors from the first model. For this analysis, males were considered to be adults from the first time they could be ranked in the dominance hierarchy (range: 14.5–16.5 years), and this dataset comprised 1965 chimpanzee-months. Females were considered to be adults from the first maximal sexual swelling for natal females (range: 9–13 years) or date of immigration, and this dataset comprised 2133 chimpanzee-months. In addition to main effects, we also examined the interactions of rank and/or reproductive state with age.

In the third set of GLMMs, we investigated time-varying covariates that were predicted to influence respiratory signs, using all chimpanzee-months. These included three climatic variables calculated on a monthly basis: rainfall, mean maximum daily temperature and mean minimum daily temperature. We also evaluated two routes of transmission: crop feeding, as a proxy for exposure to humans (other than observers); and party size, as a measure of exposure from other chimpanzees. We additionally examined dietary quality (ripe fruit consumption) to test the prediction that energy availability predicts vulnerability to respiratory illness. Prior to analysis, temporal predictors were natural log transformed, if necessary (crop feeding, party size), and then standardized using *z*-scores. To accommodate this relatively large number of variables which may be influenced by common seasonal processes, we performed multi-model inference. We began with a base model comprising the significant effects from the first GLMM, substituting the six time-varying covariates in place of the predictor, calendar month. We also evaluated a limited set of three interactions terms: rainfall × minimum temperature, rainfall × maximum temperature and crop feeding × party size (an interaction between exposure and transmission) (*N* = 130 candidate models). The best models were selected using Akaike's information criteria [35,36].

A final model considered whether clinic reports of respiratory symptoms in the nearby human community predicted respiratory signs in the chimpanzees, using data that were available for July 2008–December 2016 (102 months). Our model included the significant individual variables from the initial model, the rate of respiratory symptoms from the human community, and the number of observers contacting the chimpanzees, as well as the interaction between these two exposure variables, and the random effect of chimpanzee identity.

Linear models were run using the *glmer* function in the *lme4* package from R. Probability values were estimated with the Wald *z*-test, using the Satterthwaite approximation provided in *lmertest*. Multi-model inference and model averaging was conducted using *MuMIn.* Variance inflation factors of main effects were checked using *vif.mer* and were all below 2.5. Significance of full models and of random effects was evaluated using log-likelihood ratio tests.

## 3. Results

### 3.1. Cause-specific mortality

Of 62 recorded deaths between 1987 and 2017, 29 (46.8%) could be assigned a probable cause (table 2). Of these, respiratory illness was a factor in more than half (58.6%) of the deaths. These include 2 individuals for which necropsies were able to confirm respiratory illness as the cause of death, and 13 individuals observed with serious respiratory signs prior to their disappearance. Necropsies of two additional individuals revealed evidence of severe respiratory illness but also indicated trauma that likely contributed to death. This corresponds to a mortality rate from respiratory illness of 11.6 deaths per 1000 chimpanzee-years. Only one other individual disappeared during a respiratory epidemic, but this individual was a geriatric female and had not been observed coughing, so she was not coded as a respiratory death. Other sources of mortality were trauma—including one known and one suspected fall from a tree, one suspected death secondary to injury from a wire snare, one killed by humans and three deaths from intraspecific attacks. Five infants under the age of 5 years died after being orphaned; this included two infants whose mothers died of respiratory illness.

**Table 2.** Inferred causes of mortality for Kanyawara chimpanzees, 1987–2017.

cause (*N* deaths)	infants (<5 years)	juveniles (5.0–14.9 years)	young adults (15.0–29.9 years)	old adults (30+ years)
orphaned	5	0	—	—
respiratory illness	5	0	4^a^	7
intraspecific violence	1	2	0.5^a^	0
other trauma	2	1	0.5^a^	1
unknown	10	6	2	15
*total*	*23*	*9*	*7*	*23*

^a^Two individuals’ deaths involved severe respiratory infection and subsequent trauma and were split between categories.

Respiratory illness was the leading cause of known mortality among infants under the age of 5 years (21.7% of deaths), of young adults between ages 15 and 30 (85.7%, including two causes involving trauma) and of older adults over age 35 (30.4%). However, none of the nine deaths of juveniles aged 5–15 years could be attributed to respiratory illness (table 2).

Researchers at Gombe previously defined an ‘epidemic’ as a period of up to three months in which at least 20% of individuals exhibit respiratory signs and more than one chimpanzee dies [5,15]. Following that definition, there have been three respiratory epidemics at Kanyawara since this community began to be continuously observed in 1987. Epidemics occurred in February–March 2001, December 2006–January 2007 and March–June 2013 (table 1) and resulting in 10 total deaths. Notably, several periods of very high morbidity occurred without any deaths, and several chimpanzees died in periods that did not qualify under this definition of ‘epidemic’. The worst respiratory epidemic occurred in 2013, killing individuals across the spectrum: the alpha male, an infant, her mother, a young adult male and the oldest individual in the community (estimated age 58).

### 3.2. Frequency of respiratory signs

Of months when at least 10 chimpanzees were observed, a median of 3.4% of individuals was observed with respiratory signs per month (mean = 7.7%, s.d. = 13.1%, *N* = 248, range = 0–89.7%). In 9.3% of months, or more than once per year, at least 20% of chimpanzees exhibited respiratory signs. If only apparently unique occurrences were counted, incidence rates were 774 illnesses per 1000 chimpanzee-years. Incidence increased over time, but not significantly (*r*_S_ = 0.110, *N* = 248 months, *p* = 0.083). The year 2013 was the worst for both morbidity and for mortality.

### 3.3. Demographic predictors of respiratory signs

**Figure 1.**
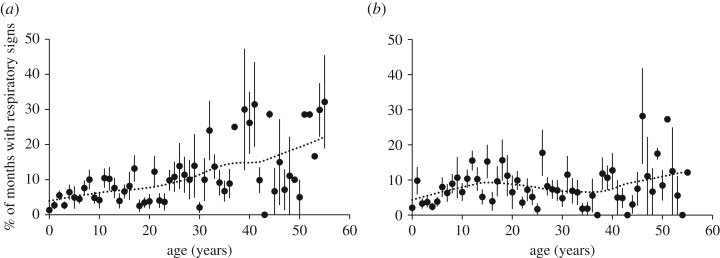
Rates of respiratory signs by chimpanzee age and sex. Shown are the means and s.e. of female (*a*) and male (*b*) chimpanzees at each age (mean of individual means per annum), excluding individuals with less than four months of observation (comprising ≥25 h per month) per year. For the figure only, ages 55 and over are combined. Data are fitted with a Loess function, *α* = 0.5.

**Figure 2.**
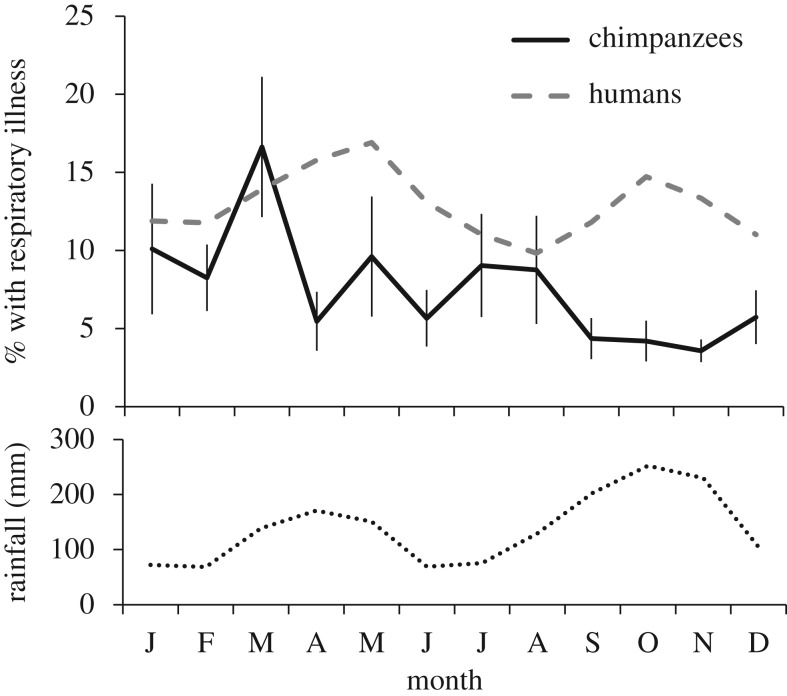
Seasonal distribution of respiratory signs. Shown in black is the mean (±s.e.) percentage of chimpanzees showing respiratory signs in each month, averaged across 22 years (1995–2016). Months were only included if at least 10 chimpanzees were observed (greater than or equal to 25 h per chimpanzee). Dashed line indicates mean monthly reports of respiratory symptoms in the adjacent human village (2008–2016). Dotted line indicates mean monthly rainfall (in mm, 1995–2016).

The first model, examining demographic predictors and comorbidities, was statistically significant compared with a null model with only the random effects (*X*^2^ = 192.6, *p* < 0.0001, table 3). Age and sex interacted to predict respiratory signs. Respiratory signs increased steadily with age in females. In males, elevated rates were observed among the oldest males (40+), but also among adolescents and young adults aged 10–20 years (figure 1). Individuals observed with diarrhoea were 63% more likely to also have shown respiratory signs in the same month, while wounds were not significantly associated with respiratory signs. Respiratory signs also varied significantly with calendar month, peaking conspicuously in March (figure 2).

**Table 3.** Results of GLMM for the monthly probability of respiratory signs among all Kanyawara chimpanzees (*N* = 8046 chimpanzee-months, 1995–2016).

fixed effects	estimate	s.e.	Wald *X*^2^ test	*p*-value
(intercept)	−4.961	0.318		
age	0.034	0.006	37.6	<0.0001
sex (M)	0.433	0.205	4.5	0.034
age × sex	−0.023	0.008	7.1	0.008
calendar month^a^	1.911	0.243	155.4	<0.0001
wound	0.138	0.155	0.8	0.373
diarrhoea	0.625	0.236	7.0	0.008
observation hours (/100)	0.430	0.079	29.8	<0.0001
signs in previous month	0.767	0.129	35.2	<0.0001

random effects	variance	s.d.	likelihood ratio test (*X*^2^)	*p*-value
chimpanzee	0.146	0.382	25.4	<0.0001
year	0.520	0.721	192.6	<0.0001

^a^Estimate reported for largest contrast, Mar versus Nov, but Wald test reports the global significance of month*.*

**Figure 3.**
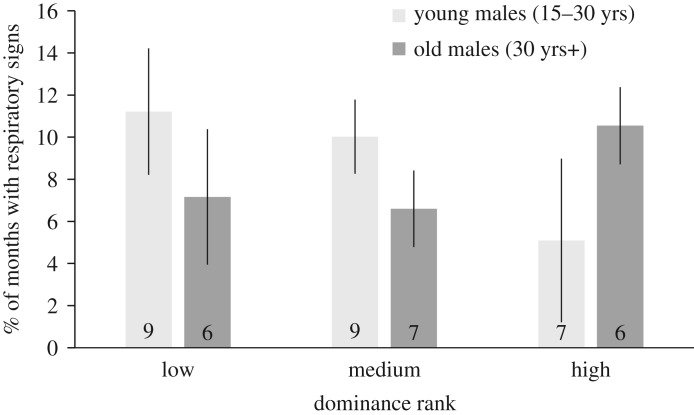
Rates of respiratory signs among adult males by age (light bars: young males, aged 15–30 years; dark bars: old males, aged 30 years+) and rank. Shown are mean ± s.e. of individual males in each category. *N* of males shown in bar, after restricting to males with at least 10 months of observation per category. Rank categories defined by dividing standardized ranks into thirds (0–0.32 = low, 0.33–0.66 = medium, 0.67–1 = high).

Among adult males, rank affected the frequency of respiratory signs in an interaction with age (full versus null model: *X*^2^ = 86.0, *p* < 0.0001, table 4). Specifically, respiratory signs were most frequent in males that were young and low-ranking or old and high-ranking (figure 3). In this model, almost no variance could be attributed to the random effect of individual, suggesting that age and rank combined are sufficient to explain inter-individual variation in the frequency of respiratory signs.

**Table 4.** Results of GLMM for the monthly probability of respiratory signs among adult male chimpanzees (*N* = 1965 chimpanzee-months, 1995–2016).

fixed effects	estimate	s.e.	Wald *X*^2^ test	*p*-value
(intercept)	−4.529	0.773		
age	−0.028	0.014	0.1	0.770
rank	−2.734	0.970	1.4	0.242
age × rank	0.082	0.032	6.7	0.010
calendar month^a^	2.824	0.577	55.9	<0.0001
diarrhoea	0.445	0.351	1.6	0.205
observation hours (/100)	0.613	0.152	16.2	<0.0001
signs in previous month	0.688	0.251	7.5	0.006

random effects	variance	s.d.	likelihood ratio test (*X*^2^)	*p*-value
chimpanzee	0.012	0.114	0.1	0.783
year	0.957	0.978	59.7	<0.0001

^a^Estimate reported for largest contrast, Mar versus Oct, but Wald test reports global significance of month.

Among adult females, neither rank nor reproductive state influenced the frequency of respiratory signs, though age was associated with an increase in signs (full versus null model: *X*^2^ = 76.0, *p* < 0.0001, table 5). None of the interactions among age, rank and/or reproductive state approached significance, so these variables were omitted from the final specification of the model. Unlike in males, significant inter-individual variation among females remained unexplained.

**Table 5.** Results of GLMM for the monthly probability of respiratory signs among adult female chimpanzees (*N* = 2133 chimpanzee-months, 1997–2016).

fixed effects	estimate	s.e.	Wald *X*^2^ test	*p*-value
(intercept)	−4.570	0.509		
age	0.034	0.011	9.2	0.002
rank	0.238	0.410	0.3	0.560
reproductive state (ref: cycling)			5.6	0.131
pregnant	0.269	0.254		
early lactation	−0.291	0.211		
late lactation	−0.148	0.249		
calendar month^a^	1.760	0.388	53.9	<0.0001
diarrhoea	−0.700	0.743	0.9	0.346
observation hours (/100)	0.392	0.141	7.7	0.006
signs in previous month	0.569	0.232	6.0	0.014

random effects	variance	s.d.	likelihood ratio test (*X*^2^)	*p*-value
chimpanzee	0.232	0.482	17.5	<0.0001
year	0.321	0.566	26.8	<0.0001

^a^Estimate reported for largest contrast, Mar versus Nov, but Wald test reports global significance of month.

### 3.4. Temporal predictors of respiratory signs

Given that calendar month was a significant predictor of respiratory signs, we investigated predictors over time: rainfall, maximum and minimum temperature, party size, diet quality and crop feeding. Many of these predictors were correlated with one another across months (*N* = 264), though none strongly (Spearman's *r* < |0.32|; electronic supplementary material, table S1). Given the potential for complex relationships among covariates, we performed a multi-model inference procedure, retaining the significant predictors from the original model (except calendar month, which we were trying to explain). The model selection process resulted in nine models within a 95% confidence set determined by the cumulative model weights (electronic supplementary material, table S2). The top four models accounted for a combined 78% probability of correctly modelling the data (table 6). Each of these top models contained all of the main effects, a significant interaction effect for rainfall and minimum temperature, and some combination of the other two interactions, which were weak and not statistically significant.

**Table 6.** Results of multi-model inference procedure on temporal predictors of respiratory signs. All candidate GLMMs (*N* = 130) included controls for individual-level predictors, as well as a random effect for subject. Shown below are model diagnostics and parameter estimates from top four models and model averaged coefficients (*β* and adjusted s.e.), and variable importance for all models within the 95% confidence set, as determined via the cumulative Akaike weights (Acc *w_I_*) (see electronic supplementary material, table S2). Also shown are the results of the top model with the variable *Calendar Month* added. Model averaged parameters (*β*), adjusted s.e. and parameter importance provided for 95% confidence set (electronic supplementary material, table S2).

model	1	2	3	4	*β*	adj s.e.	importance	1′ (w/month)
*model diagnostics*								
df	16	15	17	16				27
*Δ*AICc	0	1.79	1.97	3.75				−121.50
*w_I_*	0.403	0.164	0.151	0.062				
cumulative *w_I_*	0.403	0.567	0.718	0.780				
evidence ratio		2.5	2.7	6.5				
*candidate predictors*								
month^a^	n.a.	n.a.	n.a.	n.a.	n.a.			1.860***
party size	−0.530***	−0.570***	−0.530***	−0.571***	−0.543***	0.058	1.00	−0.539***
crop feeding	−0.406***	−0.330***	−0.405***	−0.329***	−0.385***	0.074	1.00	−0.471***
diet quality	0.231***	0.239***	0.232***	0.239***	0.231***	0.056	1.00	0.304***
max temp	0.212***	0.218***	0.211***	0.216***	0.217***	0.046	1.00	0.150**
min temp	0.072^†^	0.074^†^	0.072^†^	0.074^†^	0.068^†^	0.047	0.94	0.095*
rain	−0.091	−0.083	−0.084	−0.075	−0.073	0.058	0.86	0.001
rain × min temp	−0.113**	−0.111**	−0.113**	−0.110**	−0.093	0.057	0.83	−0.070
crop feeding × party size	0.115^†^		0.115^†^		0.081	0.072	0.71	0.081
rain × max temp			−0.009	−0.009	−0.002	0.020	0.23	
*control predictors*								
intercept	−3.482***	−3.466***	−3.485***	−3.471***	−3.480***	0.160		−4.607***
age	0.033***	0.033***	0.033***	0.033***	0.033***	0.005		0.034***
sex (M)	0.383^†^	0.382^†^	0.384^†^	0.383^†^	0.383^†^	0.201		0.395
age × sex (M)	−0.018^*^	−0.018*	−0.018*	−0.018*	−0.018*	0.008		−0.019*
diarrhoea	0.399^†^	0.416^†^	0.403^†^	0.419^†^	0.411^†^	0.227		0.471*
observation hours (/100)	0.579***	0.579***	0.580***	0.580***	0.578***	0.074		0.636***
signs in previous month	0.935***	0.929***	0.935***	0.929***	0.933***	0.125		1.013***

Significance values of parameters determined via Wald tests: ***p < 0.001, **p < 0.01, *p < 0.05, ^†^*p* < 0.10.

^a^Estimate reported for largest contrast, Mar versus Nov, but Wald test reports global significance of month.

The model selection process yielded three important insights. First, nearly all of the effects were opposite to that which we predicted. Respiratory signs increased when it was warmer, when dietary quality was higher, when crop feeding was less frequent and when chimpanzee parties were smaller. Second, despite known intercorrelations among predictors, their effects on respiratory signs were largely independent of one another. The effect size of each variable changed very little with the inclusion of other co-predictors. The only exception was rainfall, which contributed significantly only when considered in interaction with minimum temperature. Dry months were associated with increased respiratory signs only when overnight temperatures were relatively high. Third, the effect of calendar month was not mediated by any (or all) of the other covariates. When we added calendar month to the best model from the inference procedure, the model fit was markedly improved and the effects of rainfall were reduced, but the other main effects remained significant and of similar magnitude (table 6). In other words, calendar month captured all of the meaningful variation in rainfall × temperature but was not itself mediated by any of the variables we considered.

Neither the rate of respiratory infections in the nearby village (figure 2), nor the number of authorized visitors to the chimpanzees influenced the rate of respiratory signs in the chimpanzees (table 7). The interaction between the two terms was also non-significant (estimate = −0.138, *X*^2^ = 2.458, *p* = 0.117). Human respiratory symptoms peaked, on average, in May and in October, while the number of visitors peaked in January and June through August. Thus, these variables could not explain why respiratory signs in chimpanzees peaked in March. Additionally, at the time of the major 2013 outbreak in the chimpanzee community, rates of human respiratory symptoms and human visitation were no higher than normal.

**Table 7.** Results of GLMM for the monthly probability of respiratory signs among all chimpanzees in relation to exposure to human respiratory disease (*N* = 4023 chimpanzee-months, 2008–2016). The interaction visitors × human respiratory symptoms was non-significant (estimate = −0.138, *X*^2^ = 2.5, *p* = 0.117) and was excluded from the final specification of the model*.*

fixed effects	estimate	s.e.	Wald *X*^2^ test	*p*-value
(intercept)	−3.240	0.242		
age	0.031	0.009	9.6	0.002
sex (M)	0.652	0.288	2.1	0.147
age × sex	−0.022	0.012	3.0	0.081
diarrhoea	−0.308	0.406	0.6	0.449
human respiratory symptoms	−0.094	0.067	2.0	0.162
visitors	−0.101	0.062	2.6	0.104
observation hours (/100)	−0.138	0.107	1.7	0.197
signs in previous month	0.996	0.168	34.9	<0.001

random effects	variance	s.d.	likelihood ratio test (*X*^2^)	*p*-value
chimpanzee	0.255	0.505	18.5	<0.0001

## 4. Discussion

Respiratory illness is a persistent threat to the health and survival of chimpanzees at Kanyawara, accounting for more than one-quarter of all deaths and over half of deaths with identified cause. Although the cause of death for many chimpanzees remains a mystery, these data suggest that respiratory illness has been one of the primary causes, if not the leading cause, of mortality in this community. Respiratory epidemics have caused major mortality events in nearly all well-studied chimpanzee populations [1,2,4,5,8]. Respiratory outbreaks have also been observed in wild bonobos [37] and mountain gorillas, where deaths have occurred despite vigorous efforts at medical intervention [38].

While our sample is very small in epidemiological terms, the comparative mortality rates are alarming. In 2015, respiratory infections killed 37.1 per 100 000 humans globally, and 66.4/100 000 in Uganda, specifically [39]. The 1918–1920 H1N1 influenza pandemic, one of the deadliest events in modern history, is estimated to have killed approximately 500/100 000 globally, and up to 22 000/100 000 in some regions [40,41]. Using an equivalent metric, Kanyawara chimpanzees experienced an estimated mortality rate from respiratory illness of 1160/100 000 chimpanzee-years.

Respiratory illnesses in humans are most likely to affect the very young and the very old [42–47]. Consistent with the pattern in humans, we found an increase in the frequency of respiratory signs among older chimpanzees, particularly those over 40, suggesting that respiratory illness is an indicator of declining immunocompetence with age. However, in striking contrast to the pattern in humans, chimpanzee infants exhibited remarkably few signs. It is possible that coughing and other respiratory signs are more difficult to detect in clinging infants than in older individuals, but rates continue to be low even in older infants and young juveniles that are frequently off of their mothers. Infants have been targeted for focal observations since 2009, yet we did not detect more evidence of signs since this time. Despite the low rate of signs among infants, at least five infants died of suspected respiratory illness, suggesting that any given episode can be particularly lethal for these youngest individuals. All of these infants who died had mothers who exhibited respiratory signs at approximately the same time, so it is also possible that maternal health compromises contributed to mortality.

Respiratory illness was also a leading cause of mortality among both young and old adults but caused no juvenile deaths. Comparative data suggest that this is not a spurious finding (table 1). Of 72 respiratory mortality events recorded across 5 study sites, only 6 (8%) have been juveniles. Wild chimpanzee life tables [8,48,49] suggest that if deaths were to be distributed evenly across age classes, approximately 20% of deaths would be juveniles. Thus, while it has been argued that social play may be an important venue for social transmission of disease among chimpanzees [50], mirroring the impact of schools for human children, chimpanzees appear to be least impacted during the years of peak play [51,52]. The reasons for the apparent differences in human and chimpanzee respiratory epidemiology are currently unclear but merit further study.

It was surprising that respiratory illness was the leading cause of death for young adult chimpanzees, including both males and females. In contrast to humans, in which adults have acquired immunity to respiratory pathogens through repeated exposures, chimpanzees may have limited experience with some pathogens, meaning that the first exposure could pose a high risk of mortality for individuals of any age. Given that persistent exposure to human diseases is likely to be recent, chimpanzees also lack protective genetic adaptations to these particular causes [7]. Unlike humans, chimpanzees do not receive care from conspecifics to meet their basic needs when ill, so if mobility is impaired, they suffer more severe ancillary effects than would a human of the same age. For example, in several necropsies of affected individuals, the stomach and intestines were devoid of food (Kibale Chimpanzee Project, unpublished), suggesting recent inappetence or inability to feed.

Whereas the frequency of respiratory signs was high in the oldest individuals of both sexes, male chimpanzees also suffered increased signs during their late adolescent and early adult years (approximately ages 10–20). This peak coincides with male sexual maturation, including rapidly accelerating testosterone levels [53], increased muscle anabolism [54] and engagement in competition, as males enter the adult dominance hierarchy. Testosterone is predicted to have both direct and indirect immunosuppressive effects [55,56], which have been well documented in experimental studies of some vertebrates but have been difficult to demonstrate in naturalistic studies of primates [57–59]. Notably, rates of respiratory signs in young adult males were specifically increased among those lower in rank. Testosterone levels of male chimpanzees are often correlated positively with rank [52,53] (but see [54]). This suggests that high testosterone levels are probably not directly predictive of immunocompetence, *per se*, but that periods of elevated—or rapidly changing—testosterone (and attendant behavioural and physiological changes) may exact immunological costs on those less equipped to afford them.

In contrast to males, respiratory signs were not frequent among young adult females, nor did they increase in frequency among females with high reproductive costs of lactation or pregnancy. Of the eight females who died of respiratory disease, only two did so while leaving a dependent infant. Thus, despite the apparently high investment by chimpanzee mothers [60,61], there is no evidence that they were either more susceptible to acquiring a respiratory infection or more likely to die from it. Rank also did not affect the frequency of respiratory signs in females, but the random effects in our models suggest that there is additional inter-individual variation among females that we could not explain.

Our findings that individualistic factors predict rates of respiratory signs contrast with recently reported findings from Gombe, where neither age, sex, dominance rank, nor SIVcpz co-infection predicted rates of respiratory signs [16]. This may be because the 8-year period analysed in the Gombe study did not include any major respiratory outbreaks (0–9% prevalence per month). In that study, older individuals (adults versus immatures) were affected by other clinical signs, including diarrhoea and weight loss [16].

Diarrhoea and respiratory signs were significantly associated in our model considering all chimpanzees. This is notable because many instances of diarrhoea in chimpanzees are almost certainly due to diet (e.g. unripe fruits). Our data suggest that there is a significant association between diarrhoea and other indicators of health. Given the observational nature of this study, the direction of causality behind this association cannot be determined. For example, the cause of the diarrhoea could be an infectious agent that also caused the respiratory signs. Alternatively, individuals with preexisting diarrhoea (e.g. from a gastrointestinal infection or ingestion of toxins) might have been more susceptible to respiratory illness. This effect disappeared in our models of adults, so comorbidity of respiratory illness and diarrhoea was a particular risk for young chimpanzees. Similar comorbidity of diarrhoea and respiratory disease has been documented in young human children from low-income settings, with recent experience of either symptom increasing the risk of the other [62,63].

We found marked seasonal variation in respiratory signs but were unable to explain this pattern fully. Most notably, there was a pronounced peak in respiratory signs in March that was not explained by climate, diet quality or measures of exposure to humans or frequency of interaction among chimpanzees. The peak in March was not the result of just one particularly bad year. If we define an outbreak as one or more months when at least 20% of individuals exhibited respiratory signs, an unusually high number of outbreaks (6 out of 17) have occurred in March. Most notably, the factor most commonly associated with human respiratory infection rates—rainfall—had a negligible impact on respiratory signs in the chimpanzees. While March typically marked the onset of the first rainy season, there were very low rates of respiratory signs in the second, wetter rainy season in September to November. Consistent with published data from Uganda [64], respiratory symptoms in the Kanyawara village rose about a month into each wet season and, thus, did not correspond with or precede the pattern for chimpanzees. Our results also contrasted with findings from the chimpanzees at Gombe, where respiratory signs peaked in dry seasons [15].

Many of our other results were counterintuitive. We expected chimpanzees to be more often exposed to respiratory infections when feeding on human-cultivated crops and when associating with more chimpanzees, and that they would be more sensitive to respiratory illness when nutritionally stressed. Instead, we found strong and significant effects in the opposite direction. We speculate that these findings may indicate responses of chimpanzees to respiratory illness, similar to ‘sickness behaviours’ observed in other primates [65,66]. That is, once infected, chimpanzees may become less gregarious, travel less far and focus feeding on the highest-quality foods that are available. We conducted exploratory analyses to evaluate the appropriateness of a time-lagged model (i.e. one in which these factors might influence respiratory signs in the subsequent month), but no significant correlations emerged.

Neither feeding on human-cultivated crops nor the number of authorized chimpanzee observers predicted the rates of chimpanzee respiratory signs. Similarly, a study of Gombe chimpanzees found that neither the amount of time chimpanzees were followed by observers nor the time that chimpanzees spent in proximity to staff living quarters influenced respiratory signs, although provisioning with bananas did [15]. Given the links that have been made between at least some chimpanzee respiratory outbreaks and viruses of human origin, it is surprising that human factors are not of higher explanatory value. These measures are only rough proxies for human contact, so future prospective studies focused on the dynamics of disease throughout the zone of human–wildlife contact would provide important resolution on this issue. To the extent that these respiratory illnesses represent reverse zoonoses (anthroponoses), we hypothesize that respiratory outbreaks in chimpanzees are the product of low-probability transmission events that are difficult to predict, and that their severity varies with the particular pathogen transmitted and whether the chimpanzees have previously been exposed to it.

At this point, it is clear that viruses of human origin have contributed to many respiratory outbreaks in chimpanzees, including at Kanyawara [7], though we do not yet know whether there are other, more ‘natural’, causes of chimpanzee respiratory illness. Nevertheless, prevention of disease transmission should be a high priority [1,67,68]. Most chimpanzee ecotourism sites and research projects (Kanyawara included) maintain protocols to limit disease transmission, which may include quarantine periods after travel, preventing symptomatic individuals from visiting chimpanzees, limitations on visitor numbers and observation distance, wearing of face masks and disinfection of field gear [69,70]. These precautions have not been completely effective, suggesting that continued research on the epidemiology of respiratory illness in chimpanzees will be required to shape more effective conservation strategies.

Respiratory illnesses are likely to be more noticeable than other forms of disease in chimpanzees because coughing and sneezing can be heard at a distance and because respiratory illnesses often occur in distinct outbreaks. In Kanyawara, no other disease process has thus far been identified via necropsy to be a primary cause of death in a chimpanzee, and observational data have not indicated any other syndromes (e.g. gastrointestinal, integumentary) that account for more than isolated cases of self-resolving illnesses. This highlights how little we know about constraints on survival in wild chimpanzee populations. However, the relative ease with which respiratory signs can be monitored and our findings of strong patterns of individual susceptibility indicate that respiratory illness could serve as a powerful model for future investigations of immunocompetence in chimpanzees.

## Supplementary Material

Supplementary Tables

## Data accessibility

Data have been made publicly available in the Dryad repository: http://dx.doi.org/10.5061/dryad.1m004jc [71].
