# Supplementary material for: Risk factors for respiratory illness in a community of wild chimpanzees (Pan troglodytes schweinfurthii)
Source: R Soc Open Sci. 2018 Sep 19;5(9):180840. doi: 10.1098/rsos.180840 (PMC6170528; doi:10.1098/rsos.180840)
Supplement: Supplementary Tables [file rsos180840supp1.docx]

Supplementary Material for:

Risk factors for respiratory illness in a community of wild chimpanzees (*Pan troglodytes schweinfurthii*)

Melissa Emery Thompson^1,2^, Zarin P. Machanda^2,3^, Erik J. Scully^4^, Drew K. Enigk^1^, Emily Otali^2^, Martin N. Muller^1,2^, Tony L. Goldberg^5^, Colin A. Chapman^6^, Richard W. Wrangham^2,4^.

1 University of New Mexico, Department of Anthropology

2 Kibale Chimpanzee Project

3 Tufts University, Department of Anthropology

4 Harvard University, Department of Human Evolutionary Biology

5 University of Wisconsin-Madison, Department of Pathobiological Sciences and Global Health Institute

6 McGill University, Department of Anthropology

Table S1. Bivariate Spearman’s correlations between variables.

|  | | **Ordinal Month^a^ (N = 264)** | **Chimpanzee**  **Respiratory Signs^b^  (N = 248)** | **Rainfall (N = 264)** | **Maximum Temperature (N = 264)** | **Minimum Temperature**  **(N = 264)** | **Dietary Quality**  **(N = 264)** | **Party Size^c^**  **(N = 264)** | **Crop Feeding**  **(N = 264)** | **# of Observers**  **(N = 102)** | **Human Respiratory Symptoms**  **(N = 102)** |
| --- | --- | --- | --- | --- | --- | --- | --- | --- | --- | --- | --- |
| **Ordinal**  **Month** | |  | 0.11† | -0.02 | 0.17** | 0.47*** | -0.17** | 0.40*** | -0.44*** | 0.01 | -0.33** |
| **Chimpanzee Respiratory Signs** | |  |  | -0.11† | 0.13* | 0.08 | -0.01 | 0.00 | -0.19** | 0.04 | -0.01 |
| **Rainfall** | |  |  |  | -0.32*** | 0.19** | -0.04 | -0.01 | -0.08 | -0.31** | 0.11 |
| **Maximum Temperature** | |  |  |  |  | -0.06 | 0.07 | 0.14* | -0.12 | -0.02 | -0.11 |
| **Minimum Temperature** | |  |  |  |  |  | 0.08 | 0.22*** | -0.08 | 0.18† | -0.01 |
| **Dietary Quality** | |  |  |  |  |  |  | 0.18** | -0.20** | -0.16† | 0.06 |
| **Party Size** | |  |  |  |  |  |  |  | -0.21*** | 0.03 | -0.04 |
| **Crop Feeding** | |  |  |  |  |  |  |  |  | 0.18 | -0.06 |
| **# of Observers** | |  |  |  |  |  |  |  |  |  | -0.28 |
|  | ***p < 0.001; **p < 0.01, *p< 0.05, †p<0.10; ^a^Months of study numbered in order 1-264; ^b^Percentage of chimpanzees exhibiting respiratory signs, limited to months with at least 10 individuals represented; ^c^Party size was an individual measure in the models, but here is a global measure across all observations. | | | | | | | | | | |

Table S2. Results of multimodel inference procedure on temporal predictors of respiratory signs. All candidate GLMMs (N = 130) included controls for age, sex, age * sex, diarrhea, observation hours, and respiratory status in the previous month, as well as a random effect for subject. Shown below are model diagnostics, parameter estimates, model averaged coefficients ($\bar{\beta}$ and adjusted standard error, S.E.), and variable importance (Imp) for all models within the 95% confidence set, as determined via the cumulative Akaike weights (Acc *w_I_*). Also shown are the results of the top model with the variable *Calendar Month* added.

| **Model**  **Rank** | **Intercept** | **Sensitivity** | | | | | | **Exposure** | | | **Calendar Month** | **df** | **ΔAICc** | ***w_I_*** | **Acc *w_I_*** | **Evidence**  **Ratio** |
| --- | --- | --- | --- | --- | --- | --- | --- | --- | --- | --- | --- | --- | --- | --- | --- | --- |
|  |  | **Rain** | **Max Temp** | **Min Temp** | **Rain * Max Temp** | **Rain * Min Temp** | **Diet Quality** | **Crop Feeding** | **Party Size** | **Crop Feeding * Party Size** |  |  |  |  |  |  |
| 1 | -3.482^***^ | -0.091 | 0.212^***^ | 0.072^†^ |  | -0.113^**^ | 0.231^***^ | -0.406^***^ | -0.530^***^ | 0.115^†^ |  | 16 | 0 | 0.403 | 0.403 |  |
| 2 | -3.467^***^ | -0.083 | 0.218^***^ | 0.074^†^ |  | -0.111^**^ | 0.239^***^ | -0.330^***^ | -0.570^***^ |  |  | 15 | 1.79 | 0.164 | 0.567 | 2.5 |
| 3 | -3.485^***^ | -0.084 | 0.211^***^ | 0.072^†^ | -0.009 | -0.113^**^ | 0.232^***^ | -0.405^***^ | -0.530^***^ | 0.115^†^ |  | 17 | 1.97 | 0.151 | 0.718 | 2.7 |
| 4 | -3.471^***^ | -0.075 | 0.216^***^ | 0.074^†^ | -0.009 | -0.110^**^ | 0.239^***^ | -0.329^***^ | -0.571^***^ |  |  | 16 | 3.75 | 0.062 | 0.780 | 6.5 |
| 5 | -3.484^***^ |  | 0.237^***^ | 0.069 |  |  | 0.218^***^ | -0.410^***^ | -0.541^***^ | 0.104^†^ |  | 14 | 4.27 | 0.048 | 0.828 | 8.4 |
| 6 | -3.491^***^ |  | 0.230^***^ |  |  |  | 0.210^***^ | -0.410^***^ | -0.532^***^ | 0.107^†^ |  | 13 | 4.71 | 0.038 | 0.866 | 10.6 |
| 7 | -3.471^***^ |  | 0.240^***^ | 0.072^†^ |  |  | 0.225^***^ | -0.341^***^ | -0.577^***^ |  |  | 13 | 5.38 | 0.027 | 0.893 | 14.9 |
| 8 | -3.489^***^ | -0.042 | 0.224^***^ | 0.078^†^ |  |  | 0.216^***^ | -0.412^***^ | -0.540^***^ | 0.108^†^ |  | 15 | 5.45 | 0.026 | 0.919 | 15.5 |
| 9 | -3.478^***^ |  | 0.233^***^ |  |  |  | 0.217^***^ | -0.339^***^ | -0.570^***^ |  |  | 12 | 6.05 | 0.020 | 0.939 | 20.2 |
| $\bar{\boldsymbol{\beta}}$ | **-3.480^***^** | **-0.070** | **0.217^***^** | **0.068** | **-0.002** | **-0.093** | **0.231^***^** | **-0.385^***^** | **-0.543^***^** | **0.081** |  |  |  |  |  |  |
| ***Adj S.E.*** |  | **0.058** | **0.046** | **0.047** | **0.020** | **0.057** | **0.056** | **0.074** | **0.058** | **0.072** |  |  |  |  |  |  |
| Imp |  | 0.86 | 1.00 | 0.94 | 0.23 | 0.83 | 1.00 | 1.00 | 1.00 | 0.71 |  |  |  |  |  |  |
| *Top model, adding fixed effect for calendar month* | | | | | | | | | | | | | | | | |
| 1’ | -3.356^***^ | 0.001 | 0.150^**^ | 0.095^*^ |  | -0.070 | 0.304^***^ | -0.471^***^ | -0.539^***^ | 0.081 | 1.860^***^ | 27 | -121.50 |  |  |  |
| Significance values determined via Wald test: ^***^p<0.001, ^**^p<0.01, ^*^p<0.05, ^†^p<0.10, | | | | | | | | | | | | | | | | |
